# Calibración, un componente subestimado del proceso analítico en el laboratorio clínico

**DOI:** 10.1515/almed-2023-0147

**Published:** 2024-04-15

**Authors:** Oswald Sonntag, Tze Ping Loh

**Affiliations:** Consultor Independiente, Eichenau, Alemania; Consultor Senior, División de Química Clínica, Departamento de Medicina de Laboratorio, Hospital Nacional Universitario, Singapore, Singapore

**Keywords:** calibración, linealidad, seguridad del paciente, control de calidad

## Abstract

En el laboratorio clínico, la calibración de los procedimientos de medición es un aspecto clave a la hora de garantizar la fiabilidad de los resultados del paciente. A lo largo de los años, se han publicado multitud de publicaciones y procedimientos para la evaluación del control de calidad y la interpretación de sus resultados. En esta publicación, nos centraremos en un aspecto crítico, la calibración, ya que no existen publicaciones o directrices claras sobre metodologías de calibración. Por lo general, solo están disponibles las recomendaciones del fabricante del reactivo o instrumento. El propósito de esta revisión es ahondar en esta deficiencia, con el fin de suscitar un debate y mejorar la situación actual.

## Introducción

La calibración es la piedra angular de cualquier procedimiento de cuantificación, formando parte de la práctica diaria de cualquier laboratorio. En dicho proceso, se evalúa la relación entre la intensidad de señal y la concentración de un analito en el procedimiento de medida, mediante el uso de un material (calibrador) con una concentración conocida [1], [2], [3]. Tras construir la curva de calibración, la concentración de una muestra no conocida puede ser estimada sometiéndola a un procedimiento de medición y aplicando la señal obtenida a la curva de calibración para interpolar su concentración.

Con una cadena de trazabilidad adecuada, la calibración relaciona los resultados de medida de un paciente con un material de referencia o procedimiento de medida de orden superior. En condiciones ideales, se utilizaría un material de referencia primario y un procedimiento de medida de referencia primario para fijar la cúspide de la cadena de trazabilidad, lo cual es importante a la hora de estandarizar los procedimientos de medida. Sin embargo, en el laboratorio clínico, no se ha logrado generalizar el uso de dicha cadena de trazabilidad, aunque se están desarrollando iniciativas orientadas a este objetivo.

A pesar de la evidente importancia de la calibración en la generación de resultados analíticos fiables, las guías de práctica de laboratorio clínico no suelen ahondar en los procedimientos de calibración adecuados. De este modo, la principal fuente de información son las instrucciones del fabricante del instrumento de medida. El objetivo de esta revisión es profundizar en los aspectos relacionados con el proceso de calibración, escasamente abordados en la literatura científica.

## Un ejemplo de un procedimiento de medida comercial

La siguiente información contiene las típicas “instrucciones de uso” proporcionadas por un fabricante comercial de procedimientos de medida (automatizados) de bioquímica clínica.

Calibrador 1: agua destilada = blanco de reactivo

Calibrador 2: calibrador suministrado por el fabricante

Tipo de calibración: lineal

Frecuencia de calibración: calibración a dos puntos tras cambiar el lote de reactivos, y cuando los procedimientos de control de calidad lo requieran.

Trazabilidad: el método se estandarizó con un método de referencia Z, utilizándose Y como material de referencia primario.

Normalmente, se utiliza un único calibrador, realizándose además una sola medición en el procedimiento de calibración, a pesar de que la medición de calibradores también está asociada con incertidumbres de medida. El impacto de la variación asociada a la calibración se muestra a través de la siguiente serie de figuras.

**Figura 1:**
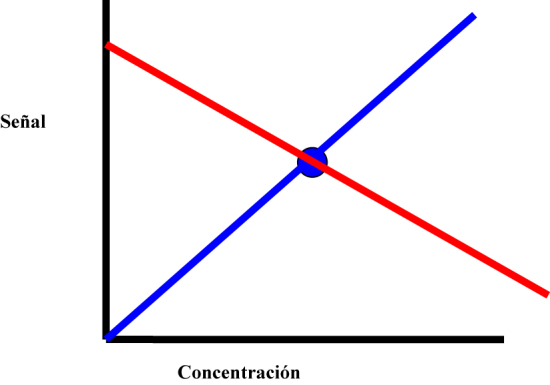
Calibración con un solo punto, donde se puede construir una regresión en cualquier dirección a través de la medición de un único calibrador. Las líneas roja y azul representan dos (de un número infinito) de las posibles regresiones de calibración negativa y positiva.

Para poder construir una regresión lineal, se requiere un mínimo de dos puntos, ya que se necesitan dos puntos para construir una línea recta. Cuando se utiliza un único calibrador, se puede construir una curva de regresión a través de la medición de un único calibrador (Figura 1), pero falta otro punto de referencia para determinar la dirección. Como resultado, no se puede inferir ninguna relación predecible entre la señal y la concentración.

**Figura 2:**
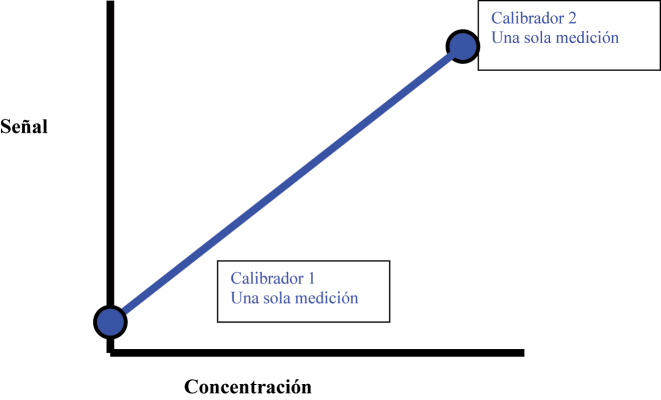
Se puede construir una regresión lineal con una calibración a dos puntos que muestra la dirección y pendiente entre la señal y la concentración del calibrador.

Cuando se realizan calibraciones de dos puntos, se puede construir una regresión lineal que describa la dirección y magnitud (pendiente) de la relación entre la señal y la concentración (Figura 2). Cuanto más compleja sea la relación entre la señal y la concentración del calibrador, más mediciones (puntos de calibración) habrá que utilizar. Por ejemplo, para construir una curva lineal, es necesario contar con al menos dos puntos, mientras que para una curva exponencial son necesarios tres puntos, precisándose cuatro puntos para construir una curva sigmoidea [4]. Cuanto mayor sea el número de puntos de calibración, mejor se caracterizará la relación entre la señal y la concentración. Es necesario emplear un mayor número de calibradores durante el desarrollo del método, con el fin de asegurarnos de que las curvas de calibración están correctamente caracterizadas antes de reducir el número de calibradores, siempre que ello no comprometa la fiabilidad de las medidas [5].

**Figura 3:**
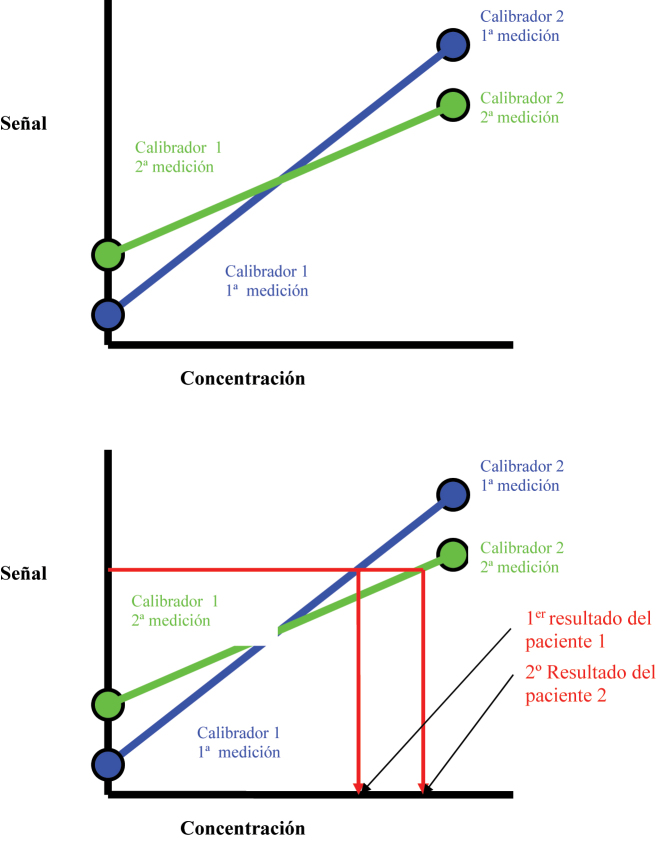
Dos curvas de calibración construidas con dos mediciones individuales diferentes de los calibradores con diferente pendiente e intersección, que se manifestarán como una diferencia en la medida observada, cuando se comparen con la misma muestra del paciente.

Además del número de puntos de calibración, otro factor importante para la fiabilidad de la curva de calibración es la variación (incertidumbre) asociada a su medición. La medición de un material de calibración (calibrador) está asociada a la misma variación analítica (y, por lo tanto, la misma incertidumbre de medición) que otras muestras de la misma matriz. Una sola medida se asocia a una mayor incertidumbre que la media de varias medidas. Cuando se utilizan los datos de una sola medida para construir la curva de calibración, esta estará sujeta a una mayor variación entre calibraciones. Esto se puede manifestar en forma de desplazamiento analítico en la medida (Figura 3).

En el laboratorio clínico, la fiabilidad de la curva de calibración se controla mediante el control de calidad interno. Esto se lleva a cabo bajo la premisa de que, en ausencia de efectos matriz clínicamente significativos, la medida de control de calidad indica que la variación entre calibraciones está dentro de los límites aceptables para informar los resultados del paciente.

En la mayoría de procedimientos de medida del laboratorio clínico, se suele utilizar un único punto de calibración en las condiciones analíticas habituales, para determinar la señal de medida del blanco de reactivo (calibrador 1) y la señal de medida del calibrador del fabricante (calibrador 2). Por cuestiones económicas, normalmente no se realizan varias mediciones de los calibradores (blanco de reactivo y calibrador del fabricante), sino que se suele confiar en las posteriores mediciones de control de calidad para identificar cualquier error grave en la calibración.

Sin embargo, esto no siempre funciona, ya que los materiales de control de calidad suministrados por el fabricante del reactivo pueden ocultar un error en la calibración. Esto puede ocurrir especialmente cuando el material empleado para el control de calidad y el calibrador son casi idénticos y se ajustan al reactivo. De este modo, existe un mayor riesgo de aceptar una curva de calibración errónea, con el consiguiente riesgo incrementado de obtener resultados erróneos en las muestras de pacientes. Dicho riesgo se puede mitigar utilizando materiales de control de calidad de terceros, tal como recomienda la norma ISO 15289:2022–7.3.7.2. [6]. “Este procedimiento también debería permitir detectar la variación entre lotes de reactivos o calibradores, o de ambos, del método de análisis”, y “se debe considerar el uso de material de control de calidad de terceros, ya sea como alternativa o como complemento del material de control suministrado por el fabricante del reactivo o instrumento”.

## ¿Qué puede hacer el fabricante del reactivo para garantizar la fiabilidad de la calibración?

De acuerdo con la Directiva de Diagnóstico *In vitro* de la Unión Europea, el fabricante debe demostrar la trazabilidad de todos los procedimientos de medida [7]. Esta trazabilidad está relacionada con un estándar o con un procedimiento de medida de referencia de orden superior. A partir de esta relación, se miden los resultados de calibración del calibrador del fabricante. Sin embargo, en los reglamentos no se establece claramente la frecuencia con la que el fabricante debe demostrar la trazabilidad (continua) del calibrador a materiales de referencia de orden superior. De este modo, a pesar de haberse demostrado una trazabilidad inicial, con el tiempo, se puede producir una desviación y desplazamiento analíticos, un sesgo analítico que se suele detectar tarde. En un estudio noruego [8], durante un período de más de siete años, se midió la concentración de glucosa en sangre en sujetos presumiblemente sanos y en pacientes con diabetes. En ambos grupos, se observó un incremento en las concentraciones de glucosa. Sin embargo, este hecho no se debía a un deterioro en el estado clínico de los sujetos, sino que estaba relacionado con cambios entre lotes del reactivo, que no fueron corregidos por el fabricante. Algunas publicaciones recientes [8, 9] han demostrado que se pueden producir diferencias significativas como consecuencia de los cambios de lote de reactivos. Por todo ello, se debe prestar especial atención a la calibración cuidadosamente. La realización de múltiples calibraciones y el empleo de material de control independiente reducen significativamente los errores, contribuyendo así a generar datos fiables de pacientes.

## Coste de los errores de calibración

En un estudio sobre los errores de calibración y la medición de calcio realizado por la Clínica Mayo, se calcularon los costos adicionales que supone no corregir un sesgo [10]. Se determinó que un error de calibración puede provocar un sesgo de entre 0,1 y 0,5 mg/dL en hasta el 15 % de las determinaciones de calcio. Además, se estimó que el impacto económico provocado por un sesgo analítico de 0,1 mg/dL oscilaría entre los 8 y los 31 dólares americanos por paciente (que se sometieron a una determinación de calcio). Teniendo en cuenta que se calcula que unos 3,55 millones de los pacientes que se someten anualmente a una prueba de calcio en suero están expuestos a un sesgo sistemático, esto se traduciría en un coste adicional de entre 60 y 199 millones de dólares al año, debido a desviaciones analíticas de 0,1 y 0,5 mg/dL, respectivamente.

## Observaciones especiales que deben tenerse en cuenta algunos factores

### Conmutabilidad, sesgo, incertidumbre de medida, trazabilidad y control de calidad

La mayoría de los estudios recientes se centran en la incertidumbre de medida [11], la conmutabilidad [9] y el sesgo con respecto al material de referencia [12]. Sin embargo, aún faltan instrucciones claras para una calibración correcta.

Miller y col [2]. afirmaban recientemente: “Un atributo esencial a la hora de lograr una trazabilidad metrológica estable es la consistencia y estabilidad de todos los elementos de la jerarquía de calibración, especialmente si se emplea una corrección de la no conmutabilidad. Cualquier cambio (por ejemplo, un cambio en la materia prima empleada en los lotes de calibradores suministrados por nuevos proveedores) debe ser evaluado cuidadosamente antes de su implementación, para evitar variaciones a largo plazo no detectadas en el sesgo de no conmutabilidad”.

En la presente revisión, no nos centraremos en los procedimientos de control de calidad [13], ya que estos se realizan para verificar la calibración, por lo que quedan fuera del ámbito de este manuscrito.

## Intento de recomendación

Actualmente, las únicas recomendaciones disponibles sobre la manera de realizar la calibración son las proporcionadas por los fabricantes de reactivos, calibradores e instrumentos. Por lo tanto, es esencial seguir estrictamente las directrices que figuran en las instrucciones de uso. No obstante, cabe señalar que muchas de dichas recomendaciones tienden a ser minimalistas por cuestiones de ahorro de tiempo y, en consecuencia, de costes. Sin embargo, es igualmente importante tener en cuenta la posible inversión de tiempo y recursos necesarios para la resolución efectiva de problemas denle los casos en los que seguir las pautas del fabricante son insuficientes. En dicho caso, debería considerarse implementar un programa intensivo de control de calidad, con el fin de mantener la fiabilidad de los resultados de las pruebas. Actualmente, existe un número razonable de programas de control de calidad publicados y en uso [13]. Por tanto, en relación a este tema, la cuestión pendiente de respuesta es: ¿Por qué destinar tantos recursos a los procedimientos de calidad de control, cuando nos estamos olvidando de la calibración?

Finalmente, nuestra recomendación es realizar primero un ajuste de cero y medir al menos dos calibradores con dos concentraciones diferentes que cubran el rango lineal, en duplicado. En el caso de ensayos no lineales, puede que sea necesario aplicar otra estrategia. La calibración se debería realizar siempre que se hagan modificaciones en el reactivo (p.e. cuando se usa una partida nueva o se cambia de lote) y/o el instrumento (por actividades de mantenimiento o reparación).

Explicación del por qué: En los ensayos de química clínica, un blanco desempeña un papel fundamental como punto de referencia en el proceso de calibración, conocido como “puesta a cero” o “ajuste inicial”. Este blanco replica todos los componentes hallados en la muestra, a excepción del analito a medir. La inclusión de un blanco es esencial a la hora de establecer una referencia inicial y desempeña un papel fundamental a la hora de eliminar el ruido de fondo y las interferencias. Esto garantiza que la señal atribuida al analito no se vea afectada por factores extraños, como las señales de la cubeta (p.ej. tras el lavado) o los reactivos (p.ej. color). Con el fin de mantener la exactitud de la medida, es habitual incluir un blanco de muestra en cada serie de muestras de pacientes analizada. Esta práctica tiene en cuenta las posibles variaciones en el ruido de fondo que puedan producirse con el tiempo o entre diferentes series de muestras, mejorando así la fiabilidad de los resultados del ensayo.

En el laboratorio clínico, es preferible realizar la calibración a dos puntos con dos concentraciones diferentes medidas en duplicado, ya que mejora la evaluación de la linealidad, aumenta la precisión, detecta y corrige errores, incrementa la robustez, y mejora la adherencia a los estándares de calidad (ISO 15189, CAP, FDA) [6, 14, 15]. Las concentraciones de los calibradores deberían abarcar el rango lineal analítico del ensayo. Esta estrategia genera resultados más fiables y de alta calidad en entornos clínicos y científicos.

Los ensayos de inmunodiagnóstico suelen tener un comportamiento no lineal, lo que requiere un enfoque distinto. Es esencial tener en cuenta las instrucciones de uso proporcionadas por el fabricante, siendo aconsejable realizar medidas repetidas para cada calibrador.

Nuestro propósito es abrir un debate sobre los procedimientos de calibración en el laboratorio clínico, haciendo hincapié en la necesidad de prestar mayor atención a los mismos, con el objetivo de ofrecer resultados fiables a los pacientes y minimizar gastos innecesarios derivados de la resolución de problemas complejos, a menudo como resultado de unos esfuerzos de calibración insuficientes.

## Definiciones a modo de glosario

Para una mejor comprensión, a continuación, se muestran las definiciones de los términos más importantes empleados en el presente estudio:

### Calibración

La calibración es el proceso mediante el cual se prueba y ajusta un instrumento o sistema de ensayo, con el fin de lograr una correspondencia entre la medida-respuesta (señal de medida) y la concentración o cantidad de la sustancia medida en la prueba. La calibración es una “instantánea” del momento, pero no permite determinar el comportamiento a lo largo del tiempo (tendencia, deriva) de un sistema de medida. Únicamente examinando varias calibraciones consecutivas, así como la información adicional obtenida de las mediciones de control de calidad, se podrán detectar tendencias y/o desviaciones.

### Calibración oficial

La calibración oficial incluye la inspección de calidad y certificación del sello de calidad. Las calibraciones son prescritas, entre otras cosas, para los dispositivos de medida utilizados en el cálculo de precios (como las básculas minoristas y los surtidores de combustible en gasolineras) y para los dispositivos médicos (por ejemplo, los termómetros para la fiebre). Dado que esta área está regulada por ley, la calibración oficial la llevan a cabo oficinas de calibración en los diferentes estados federales. El producto calibrado recibe un distintivo de verificación. El proceso de calibración debe repetirse a intervalos concretos. El término “calibración oficial” no debe confundirse con el término “calibración”.

### Ajuste

Cuando se realiza un ajuste, se modifica o regula un sistema de medida para que las desviaciones del valor determinado sean lo más bajas y conformes a las especificaciones del dispositivo. El ajuste es un proceso que altera permanentemente el sistema de medida. Suele estar estrechamente relacionado con la calibración. El objetivo de ambos procesos es detectar desviaciones, con el fin de corregirlas y documentarlas. Si estuviéramos en una situación fuera de los límites de tolerancia permisibles, se deberá ajustar la unidad, hasta que los valores medidos se encuentren dentro del límite permisible de tolerancia.

### Control de calidad

El control de calidad sirve para realizar un seguimiento continuo y documentar la calidad del proceso analítico y, por tanto, también para la verificación de la calibración. Normalmente, el control se realiza con la ayuda de material de control adecuado. En Alemania, se utilizan materiales de control con valores objetivo conocidos, siguiéndose estrictamente las directrices de la Rili-BÄK. El control de calidad debe garantizar que los resultados del análisis son fiables y se pueden utilizar tanto para fines diagnósticos como terapéuticos, lo que supone una contribución significativa a la seguridad del paciente.

### Medición

Por medición, en este caso, se entiende el proceso de determinar el grado en que un sistema de medida concreto cumple un requisito, por ejemplo, de funcionamiento o de precisión del sistema de medida. El resultado de la medición suele ser un valor medido o una serie de valores medidos. Un resultado de medición siempre es una estimación más o menos precisa con cierto grado de error de medida. El objetivo de la medición es hacernos una idea sobre un factor desconocido.

### Blanco de muestra (o simplemente blanco)

Un blanco es una muestra de referencia que no contiene la sustancia o analito de interés. Se trata de una muestra que no debería tener ninguna concentración o actividad del analito objetivo.
